
JOURNAL INFORMATION
==============================
NLM Title Abbreviation: ScientificWorldJournal
Journal ID: ScientificWorldJournal
NLM Journal ID: 101131163
Journal ID: TSWJ

The Scientific World Journal

ISSN: 2356-6140
EISSN: 1537-744X
Publisher: Wiley

ARTICLE INFORMATION
==============================
PMCID: PMC6084211
PMID: 30147517
DOI: 10.1100/tsw.2001.165
Article ID: 498680
Article version: 1
Subjects: Short Report

The Effect of Hoechst 33342 on Luciferase Gene Transcription and Translation

Zhang Xinbo
Kiechle Frederick L. *
Department of Clinical Pathology, William Beaumont Hospital, Royal Oak, Michigan 48073, USA
*Frederick L. Kiechle: fkiechle@beaumont.edu
Publication date: 2001
Electronic publication date: 2001 Nov 20
Volume: 1
First page: 54
Last page: 54
Copyright: Copyright © 2001 Xinbo Zhang and Frederick L. Kiechle.
Copyright year: 2001
License: This is an open access article distributed under the Creative Commons Attribution License, which permits unrestricted use, distribution, and reproduction in any medium, provided the original work is properly cited.
License URL: https://creativecommons.org/licenses/by/3.0/

Funding: William Beaumont Hospital Research Institute

==============================
Miami *Nature Biotechnology* Short Reports
*TheScientificWorld* (2001) 1(S3), 54SR
ISSN 1532-2246; DOI 10.1100/tsw.2001.165

### THE EFFECT OF HOECHST 33342 ON LUCIFERASE GENE TRANSCRIPTION AND TRANSLATION

Xinbo Zhang and Frederick L. Kiechle*

Department of Clinical Pathology, William Beaumont Hospital, Royal Oak, Michigan 48073
* fkiechle@beaumont.edu

**INTRODUCTION**. Hoechst 33342 (H342) and Hoechst 33258 (H258) bind to AT regions of the DNA minor groove. H342, but not H258, induces apoptosis in various cells (1). Sequence-selective DNA-binding drugs inhibit transcription factors from binding to their target sites on gene promoters *in vitro* (2). *In vivo* studies demonstrate that H342-induced apoptosis is associated with degradation of TATA box binding protein (TBP) (3) and accumulation of intracellular E2F-1 (4). Here, we determine the effect of H342 and H258 on *in vivo* luciferase gene expression in BC3H-1 myocytes using 4 pGL-3 vectors: Basic, Promoter, Enhancer, and Control.

**METHOD.** BC3H-1 myocytes were grown in MEME with 10% FBS, plated at a density of 1 x $10^4$ cells/mL, and were cultured for 2 days to 60-80% confluence prior to transfection with a pGL-3 vector using the non-liposomal formulation FuGENE™ 6 transfection reagent. After transfection for 24 h, transfected cells or non-transfected cells were treated with different concentrations of H342 or H258 (0.44 to26.7 μM) for 24 h or with 4.4 μM or 26.7 μM H342 for 24h. After the transfection and treatment, intracellular luciferase activity was measured and RT-PCR was performed.

**RESULTS.** Luciferase enzyme activity is dependent upon H342 concentration (Fig. 1). A lethal dose of H342 (26.7 μM), sufficient to induce apoptosis, suppressed luciferase activity in all four vector-transfected groups. However, H342 significantly increases luciferase activities in all four of the vector-transfected groups at sublethal doses (4.4 μM or 8.9 μM) after 24 h. All effects of H342 required a 3 hr latent period prior to initiation. Sublethal doses of H342 increase luciferase mRNA concentration.

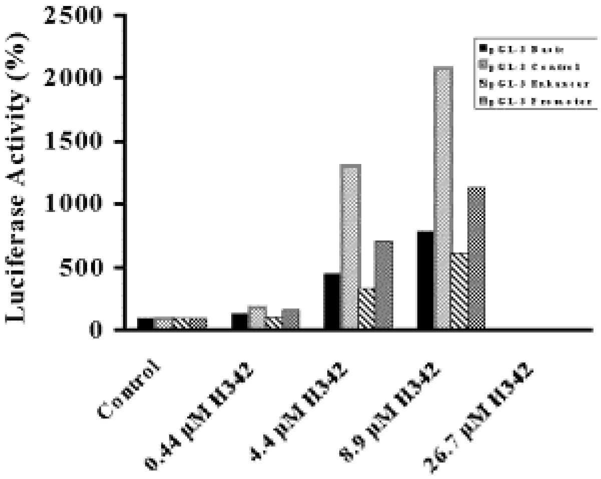

**Fig 1.** Effect of H342 on luciferase activity after transfection with 4 different luciferase expression vectors.

**DISCUSSION.** H342 inhibits the formation of TBP/DNA complexes (1,3), β-galactosidase activity in transfected Chinese hamster ovary cells (5) and c-fos mRNA in NIH3T3 cells (2). Therefore, H342 primarily inhibits transcription. In BC3H-1 myocytes transfected with four luciferase reporter vectors, H342 increased both transcription and translation at sublethal doses and suppressed them at a lethal dose. The mechanism by which sublethal concentrations of H342 increases gene expression in the absence of luciferase promoter or enhancer (pGL-3 Basic) may include an increase in formation of transcription factor initiation complex stimulated by H342 binding or an increase in cryptic luciferase promoter or enhancer binding (6) both facilitating RNA polymerase II efficiency.

**ACKNOWLEDGEMENT.** This work was supported by the William Beaumont Hospital Research Institute.

**REFERENCES.**

1. Kiechle, F.L. and Zhang, X. (2000) Arch. Pathol. 124, 804-805
2. White, C.M., Heidenreich, O., Nordheim, A., and Beerman, T.A. (2000) Biochemistry 39, 12262-12273
3. Zhang, X. and Kiechle, F.L. (1998) Biochem. Biophys. Res. Commun. 248, 18-21
4. Zhang, X. and Kiechle, F.L. Arch. Pathol. Lab. Med. (in press)
5. Werstuck, G. and Green, M.R. (1998) Science 282, 296-298
6. Thirunavukkarasu, K., Miles, R.R., Halladay, D.L., and Onyia, J.E. (2000) BioTechniques 28, 506-510
